# Mental health nursing at a time of crisis

**DOI:** 10.1111/jpm.12652

**Published:** 2020-06-01

**Authors:** Ben Hannigan, Fiona Nolan, Mary Chambers, James Turner

**Affiliations:** ^1^ School of Healthcare Sciences Mental Health Nurse Academics UK Cardiff University and Chair Cardiff UK; ^2^ School of Health and Social Care Mental Health Nurse Academics UK University of Essex and Vice Chair Colchester UK; ^3^ Faculty of Health, Social Care and Education Kingston University and St. George's University of London London UK; ^4^ Department of Nursing and Midwifery Policy and Practice Standing Group Mental Health Nurse Academics UK Sheffield Hallam University and Lead Sheffield UK

In Albert Camus' *The Plague*, the medical training of chief protagonist Dr Bernard Rieux fails to inoculate against his initial disbelief that an epidemic has arrived in the Algerian town of Oran. As Camus writes, “Everybody knows that pestilences have a way of recurring in the world; yet somehow, we find it hard to believe in ones that crash down on our heads from a blue sky” (Camus, 1947).

Thus, it has been with the novel coronavirus. Public health experts and government officials have long known of the potential for future, large‐scale, biological threat. Yet, in the UK, reports have emerged that a 2016 dry run to test the preparedness of the NHS for a pandemic produced the finding that hospitals would be overwhelmed, and supplies run short (Carrington, 2020). Early warning of the threat of the novel coronavirus (COVID‐19) came from China towards the end of 2019, and at the time of writing some five months later (the end of April 2020), the world has become an entirely different place. Millions of people across the globe have been locked down, instructed to stay indoors other than for essential journeys to prevent the virus' spread. Workplaces, schools, universities and public spaces have been deserted.

Alongside the public health effort designed to “flatten the curve” (Black, Liu, & Mitchell, 2020), early attention has focused on urgently growing capacity within acute hospital care settings to meet an anticipated surge in demand. In the UK, a network of ten “Nightingale Hospitals” to treat COVID‐19 cases only has been created with logistic support from the military (NHS England, 2020). Seven are in England, and one in each of Glasgow, Cardiff and Belfast with capacity to treat 14,980 patients in total (Ford, 2020). Implementation of the government directives outlined in Sir Simon Steven's letter of 17 March 2020 to all healthcare provider organizations in England has released the target number of 30,000 beds to treat COVID‐19 cases in existing facilities. As a result, the need for temporary COVID‐19 hospitals is drastically less than predicted with bed use of less than 100 at the time of writing (Neville, Staton, Warrell, Bounds, & Tighe, 2020).

New regulations have enabled retired health professionals to return to practice, and (to the disquiet of many in the nursing profession) to enable second‐ and third‐year student nurses to opt to work in the NHS on Agenda for Change pay grades for between 80% and 100% of their time (Nursing & Midwifery Council, 2020). Staff have been updated or retrained in critical care skills, often with support from suitably prepared nurses working in universities (for example, School of Healthcare Sciences Cardiff University, 2020).

The breadth and speed of change have been remarkable. But what of the implications of this unfolding, all‐enveloping, crisis for mental health and wellbeing and for the provision of mental health support and for professional practice? As individuals, each and every one of us is affected, including the authors of this editorial. Some of us are sleeping poorly as we think of the threats presented to loved ones and to ourselves, or find our concentration lapsing. Mild aches, pains and other ailments become reinterpreted as possible evidence of infection. News of the numbers of people dying from COVID‐19 is experienced as a daily horror, made flesh as we recall the names and see the faces of people we have known who have passed. We yearn for a return to the normal, but know that this lies months (if not years) in the future. We glimpse, too, the reality that “normal” in the period ahead may not be the “normal” we so recently lived.

As the crisis began to unfold, at the end of March 2020, Mental Health Nurse Academics UK (MHNAUK), representing educators and researchers in 70 universities, published a statement addressing six key areas and signposted readers to resources where these were available. These areas were as follows: the importance of learning from others, including from practitioners with early experience of caring for people with mental health problems and coronavirus infection; caring for self and others; mental health service responses and guidance for practitioners; recognizing the contribution of mental health nurses; supporting students; and supporting research (Mental Health Nurse Academics UK, 2020).

The MHNAUK statement observes that, in this pandemic, parity of esteem remains as important as ever. Health inequalities help explain why people with severe mental illnesses are likely to be at increased risk of coronavirus infection (Yao, Chen, & Xu, 2020). Service user‐led blogsites such as https://madcovid.com/ record the stories from lockdown of people with ongoing mental health issues and convey combinations of fear, isolation, concern at the loss of civil liberties but also of hope and strategies for coping. Others have spoken of their experiences of losing their usual face‐to‐face support, and many nurses will share concerns over the impact on vulnerable people of staff being removed from their usual responsibilities. Current COVID‐19 staffing guidance acknowledges the possibility of redeploying community mental health workers to inpatient care (NHS England & NHS Improvement, 2020), and it is in this general context that a cross‐UK project launched by the Mental Health Policy Research Unit aims to better understand how the pandemic is affecting service provision and people's experiences [https://www.ucl.ac.uk/psychiatry/covid‐19‐project].

Healthcare providers are also having to work together in different ways. For example, the mandatory situation reports submitted by all healthcare providers in England to NHS Improvement contain valuable information on COVID‐19‐related numbers of staff absences, usage of temporary staff, suspected and actual cases and deaths among staff and service users. In Wales, advice for health boards and their partners is organized through a national mental health and learning disability coordinating centre. Analysis and dissemination of data will be essential in informing service and workforce planning during the stabilization and recovery phase to come after COVID‐19.

For the present, mental health nurses working in hospitals and in the community are part of the frontline of the coronavirus response. Many will be anxious, aware of reports of critical shortages of personal protective equipment (PPE) (Mason, 2020), and of rewritten PPE guidance which has been produced with an explicit acknowledgment of a scarcity of supply (Public Health England, 2020). An independent report published on 22 April 2020 included information on 106 NHS staff known to have died, up to that date, from COVID‐19 (Cook, Kursumovic, & Lennane, 2020). Nine are described as having worked in mental health settings, and 63% of the overall number were from black, Asian and minority ethnic groups.

The Royal College of Psychiatrists has warned of a COVID‐19 crisis in UK mental health services without adequate PPE and virus testing (Royal College of Psychiatrists, 2020). Mental health nurses, like all health and social care workers, require access to protective equipment which reflects the best available evidence, along with access to accurate tests. Promises by the UK government to dramatically “ramp up” virus testing have failed, so far, to be delivered (Devlin, 2020) and many keyworking staff who have experienced coronavirus‐like symptoms are uncertain whether they have, or have not, been infected.

A rapid review by Greenhalgh et al. (2020) of the efficacy of standard face and respirator masks in preventing COVID‐type respiratory illnesses in primary care highlighted that PPE is a highly complex intervention with behavioural elements involving donning and doffing. To this might be added the complexities of the practice environment in which PPE is used, including the characteristics of the populations being served and staff members' understanding of the importance of infection control measures and their practical application.

Whilst infection control is a priority in all healthcare settings, protecting against viral infection in a critical care unit, where patients are nursed in beds, is very different from protecting against infection in mental health wards or in the home. The majority of service users in acute inpatient mental health units will be detained. Almost all are ambulatory, and many exhibit challenging behaviour. Expert groups like the UK's National Association of Psychiatric Intensive Care and Low Secure Units (NAPICU) have already produced guidance in the use of PPE and in infection control for practitioners who may be involved in physical restraint or working with disruptive behaviour in any inpatient setting (National Association of Psychiatric Intensive Care & Low Secure Units, 2020). It can be anticipated that updates will follow as experience and evidence for best practice accumulates. Risk of patient‐to‐patient and staff‐to‐patient viral transmission also needs to be contained.

In line with the Nursing and Midwifery Council (2020)’s regulations an as yet unquantified number of students have taken up band 3 or 4 NHS roles. At the start of their programmes of study, they will not have anticipated temporarily losing their supernumerary status and their protected theory time and will not have imagined being propelled in this way towards the heart of a national pandemic response. The COVID‐19 crisis has arrived at a time when the nursing workforce in the UK (Health Education England, 2019) and globally (World Health Organization, 2020) is severely depleted and has highlighted the importance of safe, sustainable and therapeutic nurse staffing in effective preparation for (and management of) future health crises. A more immediate priority is to offer support to registrants and students and to recognize the vital work that they are doing.
